# What constitutes adequate legal protection for the collection, use and sharing of mobility and location data in health care in South Africa?

**DOI:** 10.17159/sajs.2023/14605

**Published:** 2023-05-30

**Authors:** Dirk Brand, Annelize G. Nienaber McKay, Nezerith Cengiz

**Affiliations:** 1 School of Public Leadership, Stellenbosch University, Stellenbosch, South Africa; 2 Division of Law, Abertay University, Dundee, Scotland, United Kingdom; 3 Department of Public Law, University of Pretoria, Pretoria, South Africa; 4 Centre for Medical Ethics and Law, Faculty of Medicine and Health Sciences, Stellenbosch University, Cape Town, South Africa

**Keywords:** data legislation, data sharing, mobility data, location data

## Abstract

Mobile phone technology has been a catalyst that has added an innovative dimension in health care and created new opportunities for digital health services. These digital devices can be viewed as an extension of the person using them due to the deluge of personal information that can be collected and stored on them. Data collected on mobile phones are used extensively in health services and research. Personal, mobility and location data are constantly collected. The unique mobile phone architecture provides for an easy flow of data between various role players such as application developers and phone manufacturers. The collection, storage and sharing of personal information on mobile phones elicit various legal questions relating to the protection of privacy, consent, liability and the accountability of stakeholders such as health insurance providers, hospital groups and national departments of health.

## Introduction

Mobile phones have become an integral part of daily life and can be viewed as an extension of their owners given the extent of personal information collected and stored.^1^ Although initially intended for communication, mobile phones have transcended their original use and purpose to perform more versatile functions such as electronic payments, Global Positioning System (GPS) navigation, entertainment and social media applications (apps), and health monitoring.^2^ These extended functions escalate concerns about privacy and data protection as the information collected often is used by or sold to third parties.^3^

Data protection legislation largely is designed to safeguard against the exploitation of personal information through governing data collection, processing, and sharing. This protection includes data collected and processed through mobile phone use.^4^

Often data are generated and processed as an essential part of providing healthcare.^2^ The increased use and advancement of technology allow for data that would traditionally have been collected directly from patients to now be collected through mobile phones.^2^ Examples include cases of urgent medical care where real-time location is shared with healthcare professionals (HCPs) through smartphones or smartwatches and cases of remote health monitoring via digital applications that transmit data to HCPs to better bridge the barrier of access.^2^

Yet the way in which data protection legislation translates into practice, raises concerns. Are data subjects aware and adequately informed about the digital collection and processing of their personal information? How should privacy rights be managed to better protect them and legally allow for such data to be used in healthcare services?

In this article, we aim to offer guidance on the protection of privacy in the use of mobile phone data in healthcare services by addressing the above and other related questions. We include a comparative perspective about recent developments in this area in the United Kingdom (UK).

## Data collection via mobile phones

The replacement of conventional paper-based methods with digital devices has significantly improved the efficiency of data collection, storage, and sharing.^5^ The rapid pace and phenomenal scope of technological development provided by smartphones have facilitated the advanced ability to relay information on speed and direction of movement, together with visual and auditory media. This ability is fostered through the various built-in sensors and multimedia functions such as a gyroscope, digital compass, and accelerometer.^5^

Cloud service providers, developers, manufacturers and proprietors of apps, operating systems, and devices are industriously involved in the complex mobile phone landscape that includes various software layers and they serve as role players in the mobility and location data ecosystem.^5^ These role players, also referred to as responsible parties in terms of legislation, are accountable for the lawful processing of personal information that complies with the applicable data protection legislation.^6^

**Figure 1:**
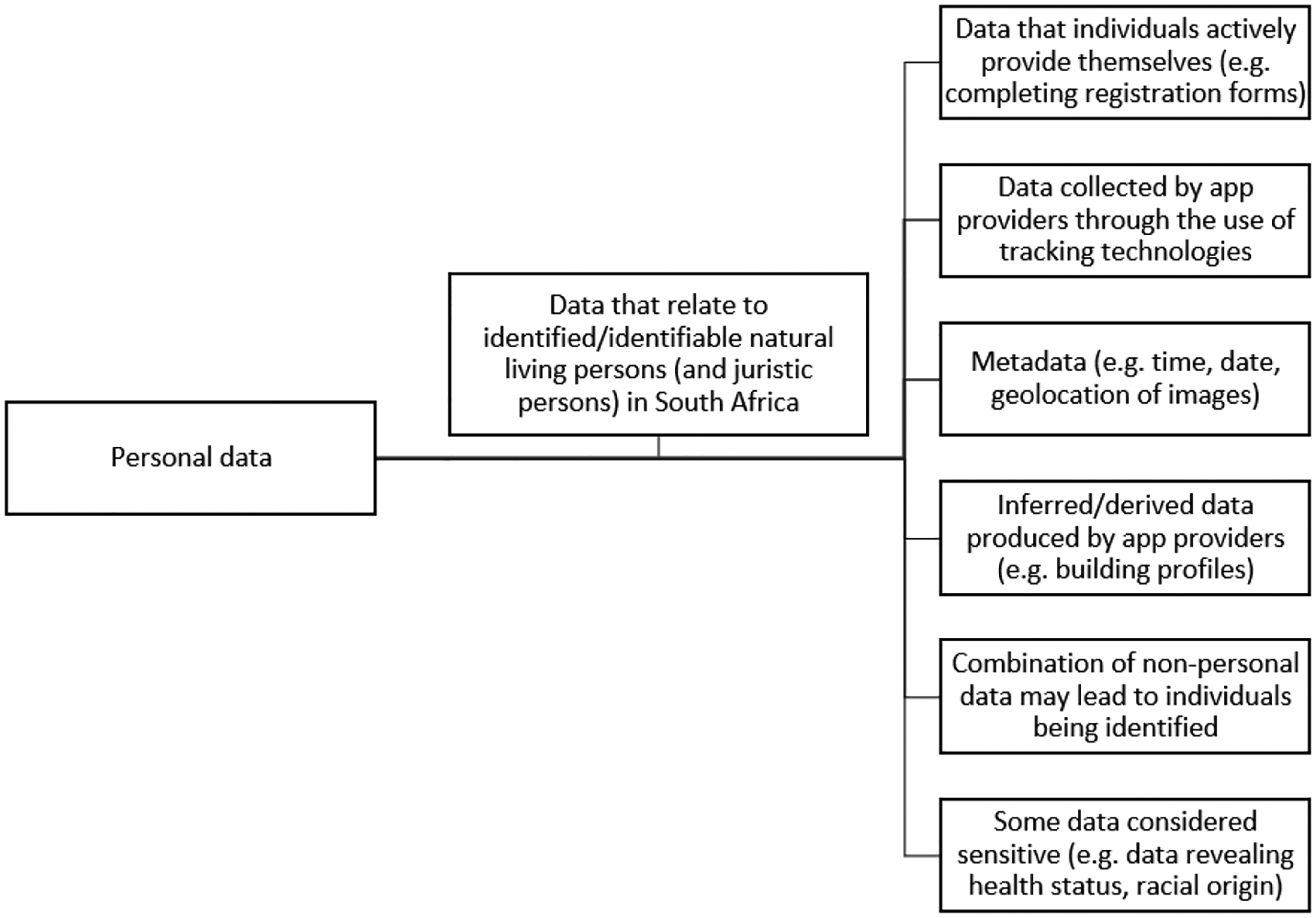
Types of data that could be considered personal data. Source: Modified from WIPO^6^ under a CC BY 4.0 licence

Section 1 of South Africa’s *Protection of Personal Information Act 4 of 2013* (POPIA)^7^ includes a broad definition of personal information which encompasses any information that can be used to identify a natural person. In the context of mobile phone users, their personal information includes location data, contact numbers, unique device and customer identifiers, credit card and payment data, telephone call logs, Internet browsing history, emails, pictures and videos, and biometric data.^8,9^ According to the European Union Agency for Cybersecurity, personal data further includes information related to the device itself, such as metadata, device identifiers and location data.^8^ Figure 1, developed by the World Intellectual Property Organization, illustrates various types of personal data that potentially could be collected by mobile devices.^6^

Although users actively collect and store such data on their mobile phones, data collection also occurs in large volumes in the background unbeknownst to the user; for example, activated device location services allow for the detection of geographical location.^6^ This capability raises questions about whether such personal information can be protected.^6^

Hence, responsible parties must ensure that users are aware of and unequivocally consent to the processing of their personal information.^9^ Consent equates to the ‘voluntary, specific, and informed expression of will’, which is a critical requirement for the lawful processing of data as indicated in section 1 of POPIA.^7^ Responsible parties must accede to appropriate data-sharing agreements.

App developers have access to the personal and non-personal data of their users and often are responsible for granting access to or selling their users’ data to third parties – data which can be used in behavioural advertising by retailer and marketing agencies.^10^ A mobile phone’s operating system is linked to various apps that provide a comprehensive set of functions to the user. Operating systems and device manufacturers have access to personal information needed to ensure smooth device and system functionality.^10^ Also, they are responsible for the application programming interface (API), which is software that enables the processing of personal information by apps on mobile devices^5^, which increases the risk of a data breach or unauthorised third-party use of personal data^10^.

The key responsibility of operating systems and mobile device manufacturers is to ensure the protection of the personal information of their users.^11^ This responsibility necessitates legally that they inform users about the processing of personal information on devices and apps and provide the users with the opportunity to opt out of any conditions or agreements relating to such processing of information.^11^ However, the manner in which the various role players or responsible parties present their privacy policies and request consent for the use and processing of personal data from users may be problematic. Problems arise often because privacy policies are lengthy and composed in technical terms, making them incomprehensible to average users of mobile phones.^11^ Complexity in the presentation of language is a violation of section 22 of the *Consumer Protection Act 68 of 2008*.^12^

Although transparency is an underlying principle of lawful data processing^6^, it is beyond the control of the individual. Often mobile phone users ignorantly or uncritically grant apps access and permission to collect and process their data where their sole purpose is to utilise the functionalities of the app in question.^13,14^

The context in which personal data are collected and the nature of the data collected are important in determining and assessing the potential risks, as sensitive information could be inappropriately integrated or contained.^13^ This possibility is because different types of data often are combined, cross referenced and used for different purposes by different role players.

Moreover, artificial intelligence (AI) is an important component at such interfaces due to its ability to use algorithms for data analysis to further link data from different apps.^14,15^ An example could be a fitness app that collects data on a user’s physical activity and connects other data from the user’s food diary app to provide an overall model of the user’s health. Thus, the integration of AI creates another layer of personal data use and risk.^14^ Analysing the use and impact of AI in health services is beyond the scope of this article, but it is important to reflect briefly on this issue as mobile phone data are fed into algorithms used to develop AI-driven products and services used in health contexts.^15^

## Use of mobile phone data and AI in health care

Health data are regarded as more sensitive than other forms of personal data, which place them higher in the level of interest for cyber criminals. Thus, this type of data receives special attention in data protection legislation such as the European Union (EU)’s General Data Protection Regulation (GDPR) and POPIA.^7,16^ Health information qualifies as “special personal information” in terms of section 26(1)(a) of POPIA, and therefore it qualifies for special protection. However, if the personal data are anonymised and cannot be re-identified, they fall outside the scope of POPIA and/or the GDPR.

In South Africa, the *National Health Act 61 of 2003* stipulates that all patient (user) information is confidential and HCPs may share or disclose that information only upon consent obtained from the patient.^17^ This requirement serves as a level of protection over patients’ personal information although the *National Health Act* is not focused on data protection as such.

As personal information collected through health- or fitness-related apps can be used by HCPs to provide healthcare services to individuals, so can digitally collected health data and even medical insurance data be used in medical research.^14,15^ According to Ventola^18^, five key categories exist for medical apps, namely “administration, health-record maintenance and access, communications and consulting, reference and information gathering, and medical education”.

In the provision of healthcare services, mobile phone data can provide up-to-date information about an individual’s state of health, which allows for remote health monitoring to better foster a HCPs clinical assessments and decision-making regarding a patient’s treatment.^15^ In remote settings or during an emergency, the use of mobile phone apps may facilitate the provision of healthcare services through obtaining immediate access to data to remotely monitor the patient’s health.^18^ By optimising the use of smartphones and health-related apps, the efficiency and value of healthcare provision may be improved through maximising time and resources. A variety of medical and health apps are available and are used in South Africa (and the UK), some of which are primarily for patients and others are aimed at HCPs. All these apps use personal data which often are combined with other data, as well as provide the services for which the app is designed. Examples of the most popular health and fitness and medical apps are provided in Table 1.^19–21^

If the personal data on a fitness or health app are sent to medical insurers or HCPs, the recipients are allowed to process that health data in terms of the exception under section 32(1) of POPIA.^7^ In the EU, the GDPR allows for such health data to be lawfully processed by HCPs and to be used in medical diagnosis and healthcare provision or treatment (Art 9(2)(h) GDPR).^16,22^

Similarly, in low- and middle-income countries where patients experience challenges in accessing health care, the use of mobile phone data enables HCPs instant access to patients’ up-to-date information.^23^ On the other hand, in high-income settings where advanced healthcare services are available, data collection through portable technological devices is essential. Smart hospitals, which are characterised by high-tech infrastructure and high-speed communication networks that “create new value and insights on patient safety, quality of care, cost-effectiveness, and patient-centeredness”, are further fostered by AI and mobile phone data.^24^

AI systems, consisting of one or more algorithms, can be used to complement the decision-making of HCPs in the diagnosis and treatment of patients.^25^ Health apps on mobile phones often operate with AI and can be utilised as a source of personal information in assessing the health of a patient. However, the training, testing and use of AI models in health care require large amounts of health data, which raises questions around the privacy and protection of patients’ personal data and, again, whether informed consent was obtained.^25^ Mittelstadt^25^ argues that these questions should be addressed on a case-by-case basis to reflect the extent to which the AI model is used to provide health care.

In addition to pertinent questions on how personal data are protected in the development and use of AI models, other important questions around the interpretability, transparency and traceability should not be ignored.^15,25^ Such questions include how AI models produce their specific output, how they are governed and what other data are required for auditing purposes? The use of AI models in the diagnosis and the treatment of patients brings into question if informed consent was obtained, or could be obtained, and, thus, impacts the doctor–patient relationship.^25,26^

## Protection of personal data concerns

When consent is requested for the processing of personal information in an app, care should be taken to ensure clarity about the purpose and scope of such processing. It is common that apps are interlinked, e.g. a fitness app that provides the possibility of sharing data on various social media apps, which increases the risk of a data breach or the unauthorised use of the personal data. In the sharing of personal data between apps, how can privacy and protection still be ensured to prevent the risk of misuse or theft by unauthorised third parties?

Mulder^22^ argues that vague language is used frequently by app providers in their statements and requests to collect and share data and, thereby, transgresses the fundamentals of informed consent and hinders the ability of individuals to provide true informed consent. This matter is cause for concern and has led to various court cases in the European Union relating to contraventions of the GDPR.^16^ In 2021, the Irish Data Protection Commission found Meta guilty of non-adherence to the GDPR’s transparency requirement to inform the users of WhatsApp of how their personal data are treated.^16^ Consequently, a fine of EUR225 million was issued.^27^

Added to the complex challenge of obtaining consent for mobile phone app use in South Africa is the low literacy levels in certain populations in the country. A study by the Department of Higher Education and Training indicates that 3.7 million adults in South Africa are illiterate.^28^ Consequently, a significant portion of the population might struggle to understand the terms and conditions of app use, let alone the implications of sharing personal health information with third parties. To address this challenge, app developers must take a user-centred approach in designing and developing apps that are easy to use and understand. Achieving this goal involves using simple language, visual aids and audio cues to convey important information to users. Also, app developers should prioritise user testing and feedback to ensure that their apps are accessible for and understandable by people with low literacy levels.

Other data protection risks in mobile phone use include the constant power-up and Internet connection which facilitate data access by unauthorised third parties. Smartphones have various sensors that collect a variety of personal data and identifiers such as the device ID, metadata, and geolocation which, together, increase the risk of tracking and user profiling without consent.^6,8^ Such collated data from different trackers installed on apps feed behavioural advertising, with users often having only limited or no control.^5,8,29^

Processing of children’s personal information receives special attention in data protection legislation such as the GDPR and POPIA, because children are regarded as a vulnerable group in society and they may be less aware of the risks involved (Recital 38, GDPR).^16^ Their personal data, for example, can be used to manipulate and influence their behaviour. A responsible party must thus take extra care when processing the personal information of children. Prior consent by a competent person, such as a parent or legal guardian, is a requirement for the lawful processing of children’s personal information (sections 34 and 35 of POPIA).^7^ These requirements apply to responsible parties in the mobile phone environment. When a mobile phone is used or an app is accessed, personal information is collected and processed, which has application to children as well. If consent is requested, it is doubtful that a competent person will always be there to provide it. If proper consent is not provided, the child’s personal information is processed unlawfully, unless another legal ground applies. Children have the same rights as adults regarding the protection of their personal information, including when they use a mobile phone.

Users of mobile phones often do not have a clear understanding of the permission required to use an app, and some apps may require more permission than is needed to function properly. This circumstance raises concerns about the legal compliance of the app providers. It is the responsibility of operating systems and app providers on mobile phones to ensure the lawful processing of personal information and, in accordance with the applicable data protection legislation, they should take extra care when the personal information of children is processed.

According to the World Intellectual Property Organization^6^, the following key principles, often found in data protection legislation, should apply to all processing of personal data in the mobile phone context: lawfulness, fairness and transparency. Application of these principles implies that:

- there must always be a legal basis for processing personal data on a mobile phone, which could be consent provided by a data subject or another legal basis specified in the relevant legislation^6^;
- processing may not lead to unfair discrimination and should avoid importing any bias^6^; and
- appropriate information about the processing must be provided in an understandable and clear way, and this could include publishing an appropriate privacy policy before installation of the app or before processing the data, and the provision of icons or privacy notifications during use of the app^5,6^.

Currently, there is no set of guidelines on mobile phone applications in South Africa. However, given the similitude between POPIA and the GDPR, the ‘Guidelines on the Protection of Personal Data Processed by Mobile Applications Provided by European Union Institutions’ may serve as guidance in our jurisdiction.^7,16^ These guidelines state that apps should collect only data that are strictly necessary for its functioning and that users must be provided with clear and accurate information to make an informed decision, with the option to withdraw their consent at any time.^30^ In Europe, the Oviedo Convention is a further legal instrument in the health context that is aimed at the protection of human rights, including the right to privacy.^31^ Article 5 of this Convention confirms the requirement of informed consent in the provision of health care to a patient.^31^

## A comparative perspective from the UK

The UK’s data protection framework predates that of South Africa, making it instructive to look at how the UK handles issues related to data privacy and the use of mobile devices to discover learning opportunities from the UK experience.

The oldest instrument in the UK’s data protection framework is an international data protection treaty to which the UK is a party, namely, the Convention for the Protection of Individuals regarding Automatic Processing of Personal Data^32^ (CETS 1981). The Automatic Data Processing Convention entered into force in October 1985 and to date has 55 ratifications or accessions.^32^ The Convention is aimed at ensuring respect for individual rights and fundamental freedoms and the right to privacy regarding automatic processing of personal data (Preamble and Article 1).^32^

The Automatic Data Processing Convention provides the data subject with rights of access to, and correction of data held by third parties (Article 8).^32^ Principles such as accuracy of data, the minimisation of data, fairness, lawfulness, and transparency in data processing are all included in the Convention (Articles 4–8).^32^ The Convention distinguishes between personal and more sensitive personal data and prohibits sensitive personal data from being processed unless appropriate safeguards are in place (Articles 5–8).^32^

In 1998, the UK enacted the *Data Protection Act* (DPA 1998).^33^ It enacted the provisions of the EU’s Data Protection Directive (Directive 95/46/EC of the European Parliament and of the Council of 24 October 1995) and was aimed at the protection, processing, and movement of personal data.^34^

In 2016, the EU enacted the GDPR 61 of 2016 (Regulation 2016 679 EU).^16^ The GDPR replaced the Data Protection Directive mentioned above.^34^ The GDPR is aimed at harmonising data processing practices and the level of data protection provided to data subjects in EU member states (Preamble, GDPR).^16^ The GDPR also applies to bodies and entities outside the EU that process data of data subjects who are in the EU (Article 3, GDPR).^16^ As the GDPR is an EU Regulation, it applies in all EU member states without the need for any further implementing or enabling legislation to be passed in those member states (an EU regulation is law once passed and published in the official journal).^16^ As the UK was a member of the EU at that time, the GDPR applied in the UK.

The GDPR’s stated aim is to harmonise data privacy laws across Europe (Article 1, GDPR).^16^ The GDPR sets out the conditions for the lawful processing of data in Article 6 and lists the conditions for the lawful consent of the data subject to the processing of personal data in Article 7.^16^ Article 8 makes provision for special conditions in the processing of children’s data, and Article 9 provides special conditions for the processing of special categories of data.^16^ Article 9(1) prohibits the processing of information related to personal data that reveals the data subject’s “racial or ethnic origin, political opinions, religious or philosophical beliefs or trade union membership, and the processing of genetic data, biometric data for the purpose of uniquely identifying a natural person, data concerning health or data concerning a natural person’s sex life or sexual orientation”.^16^ These conditions have important implications for the processing of health data on mobile phones.

Article 9(2) provides for circumstances under which the prohibition on the processing of data mentioned in sub-article 9(1) does not apply.^16^ These exclusions, inter alia, include instances where “the data subject has given explicit consent to the processing of those personal data for one or more specified purposes”; if the processing is “necessary for the purposes of preventive or occupational medicine, for the assessment of the working capacity of the employee, medical diagnosis”, or “the provision of health or social care or treatment or the management of health or social care systems and services on the basis of Union or Member State law or pursuant to contract with a health professional” (Article 9(2)).^16^

In addition, according to sub-article 9(4), member states may “maintain or introduce further conditions, including limitations”, in respect of the “processing of genetic data, biometric data or data concerning health” (Article 9(4)).^16^

However, after 31 December 2020, at the end of the Brexit transition period, the GDPR ceased to apply directly in the UK but was incorporated into UK domestic law under section 3 of the *European Union (Withdrawal) Act 2018* as well as the *Data Protection Act 2018* (DPA 2018), successor to the DPA 1998.^33^ The UK now is considered a “third country” in terms of the GDPR; nevertheless, as mentioned above, the UK’s DPA 2018 enacted the GDPR’s requirements into UK law, and closely corresponds to the GDPR.^16^ In addition, as from 1 January 2021, the Data Protection, Privacy and Electronic Communications (Amendments etc) (EU Exit) Regulations 2019 (DPPEC Regulations) that amended the DPA 2018^35^ came into effect.^36^ The DPPEC Regulations amend both the GDPR and the DPA 2018 and turn it into the UK’s new data protection framework (UK-GDPR).^16^

The UK-GDPR broadly is the same as the GDPR in terms of its substantive requirements; however, as the UK no longer is a member of the EU, it provides for an alternative enforcement mechanism.^16^ An Information Commissioner’s Office is set up as the new UK-specific supervisory body by the DPA 2018.^35^ This is an independent body which reports directly to Parliament. The jurisdiction, functions, and powers of the Information Commissioner’s Office are set out in the DPA 2018.^35^

Data privacy in the context of mobile phones in the UK is regulated further by the Privacy and Electronic Communications (EC Directive) Regulations 2003 (PECR)^37^ which implement the requirements of Directive 2002/58/EC (as amended by Directive 2009/136/EC)^37^ which provides a specific set of privacy rules for the processing of personal information by the telecommunications sector.^34^ Unlike the GDPR, the PECR remains in force in the UK despite the UK’s departure from the EU. Therefore, three main instruments or pieces of legislation constitute the UK-GDPR: the DPA 2018, the PECR, and the DPPEC Regulations.^16,35,37^

In keeping with regulations in the EU and other parts of the world, the UK-GDPR contains provisions to ensure the protection of personal data. These include the requirement that personal data be “processed lawfully and fairly”; that such processing should be based on the data subject’s consent or, if consent is absent, that it be based on another specified legal basis; it grants the data subject the right to obtain information about the processing of personal data and to demand that inaccurate personal data be rectified; it confers appropriate functions on the Information Commissioner’s Office (see above), endowing that Office with the responsibility to monitor and enforce the provisions of the UK-GDPR.^16^

Importantly, the DPA 2018 adopts the definitions of the (EU’s) GDPR, such as “personal data” meaning “any information relating to an identified or identifiable living individual”; “processing” meaning “an operation or set of operations which is performed on information, such as collection, recording, storage, disclosure, combination etc”; “data subject” as a “living individual to whom personal data relates”, and so on.^16^

On 28 June 2021 the EU adopted an adequacy decision for the UK.^38^ This means that entities in the UK that process personal data from data subjects in the EU can do so in the same way as they did previously until June 2025.^38^

On the face of it, the UK-GDPR framework constitutes a solid mechanism that protects individual privacy, including in relation to personal data being processed on mobile phones.^16^ However, research by Kollnig et al.^39^ suggests that “there has been limited change in the behaviour of cell phone apps regarding third-party tracking and the collection and sharing of behavioural data about individuals”. They state that this circumstance is a significant and ubiquitous privacy threat in mobile apps and that there exists limited empirical evidence about the efficacy of the existing EU and UK privacy protection frameworks. Specifically, Kollnig et al.^39^ found that “there has been limited change in the presence of third-party tracking in apps, and that the concentration of tracking capabilities among a few large gatekeeper companies persists”. The authors found that the GDPR has had little effect on third-party tracking across apps on the UK Google Play Store (and hence, neither has the UK-GDPR)^16,39^

A 2021 literature review by Steven Furnell, commissioned by the UK government, revealed that although, on the face of it, the UK has a watertight data privacy framework, the reality is not as clearcut as it seems.^40^ Furnell found that mobile phone app stores have “varying approaches with correspondingly variable levels of information and clarity”^40^. This variability is observed in terms of both the presence and content of their privacy and other policies, as well as in relation to supporting users’ understanding of these policies when downloading specific apps. This is particularly apparent when observing the presence and clarity of messaging about app permissions and in the handling of personal data. Some stores provide details that are comprehensive whereas others provide “nothing that most users would find meaningful”^40^.

In the light of an Australian study which found that there are significant shortcomings in relation to privacy, and inconsistent privacy practices in health-related mobile phone apps^41^, one is left wondering whether the same can be said for the UK.

## Conclusions and recommendations

In exploring the use of mobile phones in health care, this article provides an overview of the complex mobile phone landscape and identifies various legal concerns relating to the processing of personal information on mobile phones. Despite the existence of data protection legislation in most countries, the shortcomings in relation to the protection of personal information in health-related mobile phone apps identified in Australia probably are relevant everywhere.

The increased availability and use of health and fitness apps on mobile phones provide various benefits to users and HCPs. However, the risk of unlawful data processing on mobile phones still exists despite the presence of general data protection legislation. The protection of privacy on mobile phones is a challenge given a complex landscape with various role players. The most common legal basis for the processing of personal data remains the consent of the data subject. Yet operating systems and app developers often use longwinded and opaque language upon seeking consent or providing information about the purpose of data processing. This practice is of particular concern in South Africa given the low literacy level in certain population groups.

A multi-disciplinary approach – in combination with the development of clear guidance for HCPs, healthcare institutions, patients, and the manufacturers of digital devices – will address the various ethical and legal issues in digital health care. Furthermore, it is recommended that guidelines for the protection of personal data on mobile phone apps are developed based on the principles of lawfulness, fairness, and transparency. A reliance on these principles is important, not only in South Africa but everywhere. The development of legislation for the use of AI in healthcare services is recommended to further strengthen the protection of privacy and personal data in healthcare services in South Africa.

The collection, use and sharing of mobility and location data in health care in South Africa presents a scenario with significant benefits and risks. Adequate legal protection is essential to ensure that the data are collected, used and shared in a responsible and ethical manner that respects individual rights and privacy. A comprehensive legal framework that includes data protection regulations, ethical guidelines and oversight mechanisms is a necessary requirement to address the complex issues surrounding mobility and location data in health care. Such a framework should account for the unique cultural and societal contexts in South Africa. It is an imperative that policymakers, healthcare providers, and other stakeholders work together to develop and to implement an effective legal framework that protects the rights of individuals while promoting the responsible use of mobility and location data to improve healthcare outcomes. Only in doing so, can South Africa fully leverage the potential in these technologies to improve the delivery of health care and ensure that individual privacy and rights are safeguarded.

## Figures and Tables

**Table 1:** Most popular medical and/or health and fitness apps by sub-Saharan African country^19–21^

App	First ranking status (in country) on Apple App Store and/or Google Play Store	Overall downloads	Star rating
Amma: Pregnancy & Baby Tracker	Cabo Verde Guinea-Bissau Mozambique	10 M+	4.7
BetterMe: Health Coaching	South Africa	10 M+	4.1
Blood Pressure: Heart Health	Ghana Kenya Nigeria Tanzania	10 M+	4.4
Faso Santé	Burkina Faso	50 K+	4.0
Flo Period Tracker & Calendar*	Namibia Niger Mauritius Mozambique Uganda Zimbabwe	100 M+	4.6
Glow Baby Tracker & Growth App	Uganda	1 M+	4.5
HiMommy - daily pregnancy app	Nigeria	500 K+	4.7
Medscape	Zimbabwe	5 M+	4.6
Menstrual Cycle Tracker by Anastasai Kovba	Ghana	500 K+	4.7
Motivation - Daily quotes	Ghana	1 M+	4.8
Pregnancy + | Tracker App*	Niger	10 M+	4.7
Pulse - Heart Rate Monitor app	Namibia	5 M +	4.5
SICOM Health	Mauritius	500 K+	-
Smart Access!	Kenya	50 K+	
Useful healthcare apps for patients			
App	Function	Overall downloads	Star rating
Better Help	Online therapy	1 M+	3.9
MDacne	Custom acne treatment	500 K+	4.5
MySugr	Diabetes tracker log	1 M+	4.4
Teladoc Health	Telehealth and telemedicine provider (virtual care)	1 M+	4.1

* Ranked first in the medical or health and fitness apps categories.

## Significance:

We analyse the major legal concerns of mobility and location data collection and processing through mobile phones in the context of health care and provide recommendations to develop data protection guidelines that are built on the principles of lawfulness, fairness and transparency. The issues explored are of relevance in an African context and to a broader international audience.
